# Evaluating Methods for Detrending Time Series Using Ordinal Patterns, with an Application to Air Transport Delays

**DOI:** 10.3390/e27030230

**Published:** 2025-02-23

**Authors:** Felipe Olivares, F. Javier Marín-Rodríguez, Kishor Acharya, Massimiliano Zanin

**Affiliations:** Instituto de Física Interdisciplinar y Sistemas Complejos (CSIC-UIB), Campus UIB, 07122 Palma, Spain; felipe@ifisc.uib-csic.es (F.O.); fco.javier.marin.rdz@gmail.com (F.J.M.-R.); kishor@ifisc.uib-csic.es (K.A.)

**Keywords:** time series, stationarity, functional complex networks, ordinal patterns, causality

## Abstract

Functional networks have become a standard tool for the analysis of complex systems, allowing the unveiling of their internal connectivity structure while only requiring the observation of the system’s constituent dynamics. To obtain reliable results, one (often overlooked) prerequisite involves the stationarity of an analyzed time series, without which spurious functional connections may emerge. Here, we show how ordinal patterns and metrics derived from them can be used to assess the effectiveness of detrending methods. We apply this approach to data representing the evolution of delays in major European and US airports, and to synthetic versions of the same, obtaining operational conclusions about how these propagate in the two systems.

## 1. Introduction

In the past few decades, functional complex networks have emerged as a powerful tool for the analysis of the dynamics of real-world systems. Many of these are complex, in the sense of being composed of multiple elements interacting in a non-linear way, such that the global dynamics cannot be inferred by the behavior of the individual constituents [1]. At the same time, these interactions are seldom observable; hence, the problem is how to describe the structure created by leveraging only limited information. The solution often entails reconstructing complex networks [2], where nodes represent the individual elements of the system and links between pairs of nodes are established whenever their dynamics are shown to be connected. In other words, the dynamics of the elements are assumed to be a function of these connections, and detecting the latter from the former becomes an inverse problem. This approach has been applied in multiple scientific fields, spanning from neuroscience [3,4,5] and genetics [6,7,8] to climate [9,10]. It has more recently been used to unveil the patterns behind the propagation of delays in air transport [11,12,13], a problem impacting the cost-efficiency and safety of the system, and with an important negative impact on the environment [14,15]. In general terms, the analysis is conducted by extracting time series representing the evolution of delays at each airport and then applying a statistical test evaluating the presence of correlations or causalities between pairs of time series. The results of these tests can also be represented as a complex network, allowing more detailed topological analyses [2,16,17].

One of the fundamental prerequisites for obtaining representative functional networks is the stationarity of the time series used in their reconstruction. From a statistical perspective, a time series is said to be stationary when its properties do not have an explicit dependence on time and thus has no predictable patterns—e.g., there must be no trends or seasonalities. Please note that any measure averaged over a non-stationary time series will not be accurate and that standard tests (including the *t*- and *F*-tests) and models (like the Auto Regression Moving Average, ARMA) may yield incorrect results. Intuitively, time series representing the evolution of delays at a given airport are expected to be highly non-stationary; delays are usually correlated with the traffic volume, such that they will be higher at noon and lower at night and early morning. This creates regular oscillations that can be misinterpreted as correlations, or even causalities, between pairs of time series.

**Figure 1:**
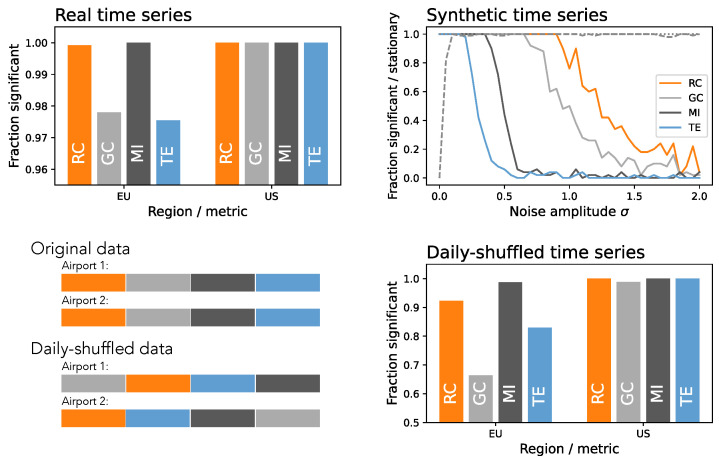
Nonstationarity and its consequences in the estimation of functional connectivity. (**Top left**) Fraction of pairs of time series for which functional connectivity is detected, for Europe (left bars) and the US (right bars), and four different connectivity metrics. See Section 2 for details on the data and methods. (**Top right**) Fraction of times a functional link is detected between the time series of Paris Charles de Gaulle and London Heathrow as a function of the amplitude of a Gaussian additive noise of zero mean and standard deviation σ. Please note that the amplitude is normalized, such that σ=1 corresponds to the standard deviation of the time series. The dashed and dotted gray lines correspond to the fraction of times the time series are detected as stationary by, respectively, KPSS [18] and Augmented Dickey–Fuller [19] tests. (**Bottom left**) Graphical representation of the creation of daily shuffled surrogate time series; each color represents the data of one day. (**Bottom right**) Fraction of pairs of time series for which functional connectivity is detected when daily shuffled surrogates are used. The meaning of bars and colors is the same as in the top left panel. In all cases, RC: Rank Correlation; GC: Granger Causality; MI: Mutual Information; TE: Transfer Entropy.

To illustrate this latter point, the top left panel of Figure 1 reports the fraction of pairs of airports for which metrics detect a flow of information for airports in Europe and the US. Details about the data and the four metrics are presented in Section 2 below. Results indicate that the propagation of delays forms an all-to-all complete network. While not impossible, this result has been obtained using a raw time series of delays and may thus be the consequence of their nonstationarity. Interestingly, when applying two classical stationarity tests, i.e., the Kwiatkowski–Phillips–Schmidt–Shin (KPSS) [18] and the Augmented Dickey–Fuller [19] unit root tests, only the former detects the presence of regular trends. Additionally, adding a small amount of observational noise confounds both tests, while the four metrics still yield a large fraction of causality links—see top right panel of Figure 1.

In order to further validate the role of stationarity, we create surrogate time series by maintaining the same values observed within each day but randomly shuffling the days in each time series—see the bottom left panel of Figure 1 for a graphical representation. In these surrogate series, values at the same position will correspond to delays at the same time but on different days; hence, any detected relationship must be due to the trends within them and not to true propagation instances. As reported in the bottom right panel, many relationships are still detected. In short, ways to detrend these time series and to evaluate the final performance are imperative, in order to obtain reliable functional networks.

Looking at the results of Figure 1, one may be tempted to use these daily shuffled time series as a benchmark—i.e., if the number of detected links is low, it may be concluded that the detrending method is acceptable. This is, nevertheless, not a universal solution, as it depends both on the metric used to assess relationships and on the data. Again, referring to the bottom right panel of Figure 1, it can be appreciated that the Granger Causality (GC, light gray bars) yields substantially different results to the Mutual Information (MI, dark gray bars), even for the same data; and results are always worse for the US, probably due to the greater length of the time series. Consequently, when using GC on the European data, one may be misled to believe that the data are (at least partially) already detrended; but this would result in major biases when considering other metrics or datasets. In other words, obtaining few functional links with a given metric does not guarantee that the underlying time series are truly stationary; false connections may be found in different conditions.

In this contribution, we tackle the problem from an alternative perspective. Given a set of detrending methods, we ask whether independent metrics can be used to assess the number of temporal patterns in the detrended time series. The underlying hypothesis is that whenever these metrics detect no such patterns, the detrending process is effective, and hence, no spurious functional connections should be detected. Conversely, if patterns remain, the time series could not be assumed to have been effectively detrended, and the obtained functional connectivities should, therefore, not be trusted. Among the many possibilities available in the literature, we tackle this problem using ordinal patterns, i.e., a symbolic representation of time series values focusing on the relative amplitude of neighboring data points [20,21]. These patterns then allow the description of the serial dependence structure present in the studied time series in a simple way while providing advantages that include easy interpretability and low computational cost [22,23]. Two versions of the same are compared—respectively, a traditional (see Section 2.2.2) and a continuous one (Section 2.2.3), the latter being able to better incorporate amplitude information [24]. As previously hinted, we test this hypothesis using data representing the evolution of delays at major European and US airports (see Section 2.4) and synthetic versions of the same, with the aim of describing propagation patterns between them. The results presented in Section 3 depict a complex scenario in which detrending methods commonly used in other contexts here underperform. Conversely, when knowledge about the underlying system is included, highly stationary time series can be obtained, which can be used to extract interesting conclusions about the delay propagation process—as discussed in Section 4. In short, we here demonstrate that ordinal patterns-based metrics can provide a model-free methodology to evaluate and validate detrending algorithms.

## 2. Materials and Methods

In this section, we are going to introduce the methods and data considered in this work. Following the structure of the analysis, these will include the algorithms for detrending the data (see Section 2.1), the metrics to evaluate the correctness of the detrending process (Section 2.2), and the procedure for reconstructing functional connectivity (Section 2.3). We finally introduce the real air transport data on which the methodology will be tested in Section 2.4.

### 2.1. Detrending Methods

In order to remove the trends present in the considered time series, we here use some standard methods, both representing standard statistical approaches and developed using the knowledge of the problem at hand—specifically, the fact that delays have daily and weekly trends [25].

- Identity (*Ident*). As a reference, in the following analyses, we include the results corresponding to not performing any detrending on the time series.
- Delta (*Delta*). Simple detrending process based on evaluating the distance between the value at a given hour and the expected value observed for the same weekday and at the same hour: $\Delta x\left(t\right)=x\left(t\right)-\langle x(t+24\cdot 7\cdot k)\rangle$, with k∈Z and $\langle \cdot \rangle$ representing the average.
- Independent Component Analysis (*ICA*). The time series are detrended by subtracting the three main components detected throughout all the airports by an Independent Component Analysis [26]. While computationally more costly than other solutions, it presents the advantage of being able to detect trends with variable periods, provided they are shared by multiple airports.
- Second Derivative (*SecD*). The second difference of the time series, i.e., $x^{\prime \prime }\left(t\right)=x\left(t\right)+x\left(t-2\right)-2x\left(t-1\right)$. This approach is customary in the literature when no information about the nature of the underlying periodic trends is available.
- Z-Score by day (*ZScore24*). Detrending based on a Z-Score, defined as: $z\left(t\right)=\left[x\left(t\right)-\langle \tilde{x}\left(t\right)\rangle \right]/\sigma_{\tilde{x}\left(t\right)}$. $\tilde{x}\left(t\right)$ represents the set of values observed on different days at the same hour, i.e., x(t+7·k) with k∈Z. In turn, $\langle \cdot \rangle$ and $\sigma_{\cdot }$ represent, respectively, the average and the standard deviation operators. The Z-Score encodes how much the observed value deviates from the expectation, in this case from the delay observed at the same hour on other days; but, as a difference with respect to the Delta approach, it takes into account the variability of the data.
- Z-Score by week (*ZScore724*). Same as the ZScore24, but taking as reference the delays observed at the same hour on the same day of the week. $\tilde{x}\left(t\right)$ is thus here defined as x(t+24·7·k) with k∈Z. ZScore724 should, therefore, also detrend with respect to weekly patterns, e.g., weekdays vs. weekends.

### 2.2. Evaluating the Detrending Process

As outlined in the introduction, the quantity of information contained in the time series is here assessed through two metrics, the JSD and the COP, both based on the application of the concept of ordinal patterns. In what follows, we first introduce the latter one and then define the two metrics.

#### 2.2.1. Ordinal Patterns in Time Series

Bandt and Pompe introduced an encoding scheme that maps a raw time series onto a corresponding sequence of symbols called ordinal patterns [27]. This is simple, model-free, and with strong resilience to noise [28,29,30]. Several applications pioneered by Prof. Osvaldo Rosso have confirmed the success of this symbolization technique in the evaluation of information quantifiers [31,32,33]. The existence of forbidden [28,34] and missing ordinal patterns [35,36] have been applied to different areas from finance [36,37], semiconductor lasers [38], atmospheric turbulence [36], chaotic optoelectronic systems [39], and hydrology [36]. Furthermore, ordinal patterns have been used to quantify persistence [40], symmetry [40], irreversibility [41,42], and serial dependences [22,23] in time series.

Given a time series $X\left(t\right)=\left\{x_{t};t=1,\ldots ,M\right\}$, sub-windows (or segments) of it, composed of *D* values, can be mapped into vectors. Specifically, *D* consecutive (τ=1) or non-consecutive (τ>1) values starting at time *t* can be transformed into a vector $\left(x_{t},x_{t+\tau },\ldots ,x_{t+(D-1)\tau }\right)$ of dimension *D*. Each element of the previous vector is then replaced by its relative ranking, from zero for the smallest value up to D−1 for the largest one. Next, this vector is itself encoded as an ordinal pattern, representing the permutation $\pi_{i}$ of 0,1,…,D−1 describing the relative amplitude (strength) of each element in the considered sub-window. Finally, all ordinal patterns, calculated for all possible sub-windows, are synthesized into a probability distribution $\#(\pi_{i})$, usually normalized by the total number of ordinal patterns M−(D−1)τ. Please note that the condition M≫D! must be satisfied in order to obtain reliable statistics [27].

**Figure 2:**
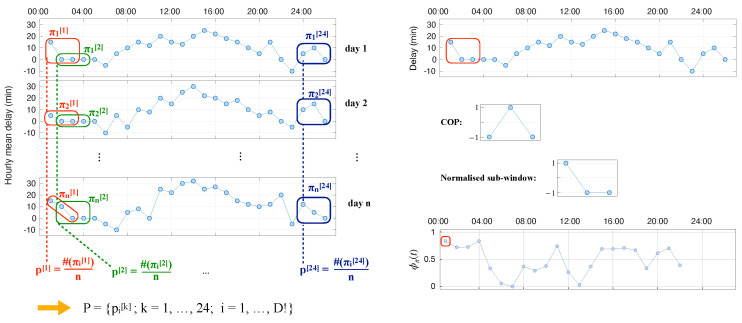
Graphical representations of the two methods for evaluating the detrending process. (**Left**) Illustration of building the ordinal pattern distribution over *n* days of hourly mean delay sequences. Red and green boxes indicate the data used to calculate the first two distributions. (**Right**) Calculation of the $\phi_{\pi }$ for a time series of delays using the COP methodology. See main text for details.

To illustrate how we build the ordinal pattern probability distribution in this study, let us consider an example comprising a synthetic hourly mean delay sequence over *n* days, as shown in the left panel of Figure 2. For a pattern length D=3, we extract a set of ordinal patterns for each day, representing the ordinal information of the first *D* hours. After normalizing by *n*, a probability distribution is obtained. By repeating this procedure with sliding windows of length *D* and overlap of D−1, we can generate a set of 24 distributions representing the ordinal probabilities of each hour of the *n* days. Please note that for the 24th hour, we consider the 2 h of the following day. We follow the recipe suggested in [27] for breaking ties by adding a small amount of Gaussian noise (zero mean). This latter ingredient is important due to the inactivity periods, for which the hourly mean delay equals zero for several consecutive hours.

#### 2.2.2. Jensen–Shannon Divergence of Ordinal Patterns

The permutation Jensen–Shannon Distance (JSD in short) of ordinal patterns was recently introduced as a versatile metric for assessing the degree of similarity between the symbolic ordinal sequence statistics of two time series [43]. This metric relies on evaluating the similarity between the ordinal pattern probability distributions $P=\{p_{1},\ldots ,p_{N}\}$ and $Q=\{q_{1},\ldots ,q_{N}\}$ associated with the two time series under analysis, using the Jensen–Shannon divergence [44], providing a quantitative measure of their statistical resemblance,

(1) $$D_{\mathrm{JS}}\left(P,Q\right)=S\left(\left(P+Q\right)/2\right)-S\left(P\right)/2-S\left(Q\right)/2,$$

where $S\left(P\right)=\sum_{i=1}^{N}p_{i}\ln p_{i}$, is the classical Shannon entropy. The JSD is hence obtained by calculating the square root of Equation (1), and its normalized version reads

(2) $$\mathrm{JSD}\left(P,Q\right)=\sqrt{\frac{D_{JS}\left(P,Q\right)}{\ln2}}.$$

Higher values of the JSD suggest greater dissimilarity between the symbolic representations of two time series, while lower values indicate closer similarity. Intuitively, signals originating from the same underlying dynamics are expected to yield small ordinal distance values approaching zero but not exactly zero due to finite-size effects [43]. Additionally, it allows for easy hypothesis testing regarding the dynamical nature of any given time series by calculating its JSD relative to reference time series generated in alignment with a null model or surrogate sequences [43,45].

In the present analysis, we calculate the JSD between each of the 24 ordinal pattern distributions (one per hour, as illustrated in Figure 2) and their shuffled surrogates. These randomized sequences are constrained realizations that satisfy the null hypothesis, i.e., stationary and absence of temporal correlations across days. Moreover, in such a way, finite-size and amplitude distribution effects are taken into account. Finally, to obtain one value for each airport we average the JSD over the 24 h.

#### 2.2.3. Continuous Ordinal Patterns

Continuous Ordinal Patterns [24] (COPs) are a recently proposed modification of the original idea of ordinal patterns previously described, in which the output, instead of being discrete (i.e., a single ordinal pattern), is continuous [24]. Specifically, given a COP and a sub-window of the time series, this approach entails calculating the distance between both. When compared to classical ordinal patterns, this yields the advantage of naturally including the (local) amplitude information of the time series under analysis. On the other hand, the pattern moves from being an output of the analysis to being an input of the same. A brief discussion of the COP methodology is reported here for the sake of completeness; the interested reader can find additional details in [24].

We start by defining a continuous ordinal pattern π as a set of *D* values $\pi =(\pi_{0},\pi_{1},\ldots ,\pi_{D-1})$ normalized in the range [−1,1]. *D* is here the embedding dimension, and it has the same meaning as the embedding dimension of ordinal patterns. We further denote by $s^{*}$ the sub-window of length *D* of the original time series *X* that is currently under analysis; note that this segment is also normalized in the range [−1,1]. Given both π and $s^{*}$, we can then define a distance $\phi_{\pi }$ assessing how well the former represents the evolution of the data in the latter:

(3) $$\phi_{\pi }=\frac{1}{2D}\sum_{i=1}^{D}d_{i}=\frac{1}{2D}\sum_{i=1}^{D}\left|\pi_{i}-s_{i}^{*}\right|.$$

Here, the subscript *i* denotes the *i*th element. Please note that $\phi_{\pi }=0$ implies that $s^{*}$ and π are exactly equal, or, in other words, that the pattern π is a perfect representation of the dynamics within the sub-window $s^{*}$. On the other hand, values of $\phi_{\pi }$ close to one imply that $s^{*}$ and π have substantially different dynamics. $1-\phi_{\pi }$ is then a metric assessing how well the pattern π represents the dynamics of the time series, and this can further be averaged over all possible segments of length *D* of the analyzed time series to quantify how important π is to understand its dynamics. Hence, the larger the value of $1-\phi_{\pi }$ (and the smaller is $\phi_{\pi }$), the more the pattern π is present in the data.

Please note that, up to this point, the COP π has been given as an input of the analysis; it is then necessary to define a way of finding the best π for the problem at hand. In this study, we resort to a simple strategy previously proposed in Refs. [24,46], and based on testing a large set (here, 250) of random patterns π. For each test, the distance between the values $\phi_{\pi }$ obtained in the original time series and those in a randomly shuffled version of the same is estimated through a Kolmogorov–Smirnov two-sample test. Finally, the π yielding the largest difference, and thus the pattern according to which the time series under analysis is farther away from a random sequence, is retained.

In synthesis, given a single time series *X*, the full process involves: (i) finding a COP of dimension *D* for which the distance between *X* and a randomly shuffled version of it is maximal by testing multiple random COPs; (ii) calculate the median of $\phi_{\pi }$ using such COP; finally, (iii) the maximum $\phi_{\pi }$ obtained across the multiple COPs (in what follows denoted as COP for simplicity) is understood as an index for the presence of some non-trivial structures in the time series. The whole process is illustrated in the right panel of Figure 2, representing, from top to bottom: the original time series *X*; an arbitrary COP of size D=3; the extraction and normalization of the first sub-window, also of length D=3, and corresponding to the red box; and finally, the calculation of the complete $\phi_{\pi }$, in which the first value corresponds to the previous sub-window.

#### 2.2.4. Metric Normalization

In the previous sections, we have defined two ways of detecting the presence of patterns in time series and, hence, whether they can be considered random. Understanding these metrics entails an additional challenge: while they measure a distance from randomness, a completely random time series would also give positive distances due to finite-size effects. In order to solve this, both the JSD and the COP calculated from a given time series are here normalized according to the average values obtained by the same metrics in a large ensemble of surrogate (randomly shuffled) time series. In other words, the original time series are randomly shuffled 50 times; the JSD and the COP are calculated for each one of these surrogates and averaged, respectively, obtaining $\langle JSD_{rnd}\rangle$ and the $\langle COP_{rnd}\rangle$; finally, normalized versions of the metrics are defined as $JSD^{*}=JSD/\langle JSD_{rnd}\rangle$ and $COP^{*}=COP/\langle COP_{rnd}\rangle$. Those normalized values will have an expected value of 1 in the case of random time series and greater than 1 whenever non-random structures are present.

To further validate the obtained results, we have also considered an alternative normalization method based on using both the average and the standard deviation of the values obtained in the surrogate time series to define a Z-Score; in what follows, these two will be, respectively, denoted as $JSD^{Z}$ and $COP^{Z}$. This provides a more precise quantification of the distance from randomness, especially in the case of short time series, for which the variability both in the ordinal pattern frequencies and in the COP is higher. The length of the time series considered in this study (see Section 2.4) is large enough to not impact the results, and the relationship between $JSD^{*}$ and $COP^{*}$ and their Z-Score counterparts is almost linear. Still, for the sake of generalizability, both normalizations will be evaluated below.

### 2.3. Assessing Functional Connectivity

Once the time series have been detrended, the next step in the analysis involves the detection of functional connectivity between pairs of them or, in the context of the present study, the detection of instances of delay propagation. Thus, given two time series *X* and *Y* for which we want to detect connectivity X→Y, the analysis is conducted by applying the following four functional tests:

- Rank Correlation (RC). Spearman’s Rank Correlation between the two analyzed time series, calculated over shifted time series x(t),y(t+λ), with λ∈{0,1,…,5}, to account for the time required by delays to propagate. The λ yielding the lower *p*-value is the one selected.
- Granger Causality (GC). The GC [47] is one of the best-known exponents of *predictive causality* [48] and assesses whether the inclusion of information about the driving element *X* helps predict the future dynamics of the driven element *Y*. As originally proposed, an autoregressive-moving-average (ARMA) model is used for the prediction. Two variants are constructed, forecasting *Y* by, respectively, introducing or not data about *X*’s past. Finally, the two models’ residuals are compared through an F-test, yielding a *p*-value indicating whether the presence of information about *X* is relevant—and, hence, whether a causality relationship is present.
- Mutual Information (MI). MI is an information-theoretic measure that captures the shared amount of information between any two random variables. The Shannon information for *X* and *Y*, respectively denoted as H(X) and H(Y), represent the corresponding amount of potential information or the degree of uncertainty [49]. MI quantifies how much of the uncertainty in *Y* is reduced or explained after knowing the full information of *X*, i.e.,
  (4) $$I\left(X:Y\right)=-\sum_{y}\sum_{x}p\left(x,y\right)\log\left(\frac{p(x,y)}{p(x)p(y)}\right),$$
  where p(x,y) represents the joint probability distribution, while p(x) and p(y) denote the marginal probability distributions. MI is particularly interesting for investigating the relationship between two variables because, in contrast to cross-correlation, it is sensitive to non-linear dependencies. In this work, MI is estimated using the Kozachenko–Stögbauer–Grassberger (KSG) method [50], which is well suited for continuous random variables following non-parametric (or unknown) distributions.
- Transfer Entropy (TE). TE is also an information-theoretic measure that captures the amount of directional information flow from a source variable (e.g., *X*) to a target variable (e.g., *Y*) [51]. It is the measure of the amount of information contained in the past states of a source process (i.e., $X^{-}$) about the future state of the target process (i.e., *Y*) given that the past states of the target (i.e., $Y^{-}$) are known:
  (5) $$TE_{X\to Y}=\sum p\left(y_{n},y_{n}^{-},x_{n}^{-}\right)\log\left(\frac{p(y_{n}|x_{n}^{-},y_{n}^{-})}{p(y_{n}|y_{n}^{-})}\right).$$
  TE is a model-free directional measure that captures the casual relationship in Wiener sense between two variables [52]. The KSG algorithm developed for MI has been adopted to compute the TE [53], and the same adaptation has been used in this work.

Please note that the result of applying these four tests is a *p*-value—in the case of the MI and the TE, this is obtained by comparing the measured information to what is obtained in a set of $10^{3}$ surrogate (randomly shuffled) time series. As a final step, the presence of a propagation link between two airports is accepted whenever such *p*-value is below a significance threshold α=0.01.

### 2.4. Data on Airport Dynamics

In order to validate the analysis here proposed on a real-world scenario, we consider two complementary data sets describing the hourly evolution of delays in the top-50 airports in Europe and the US. Information has, respectively, been obtained from EUROCONTROL’s R&D Data Archive, a public repository of European historical flights made available for research purposes and freely accessible at https://www.eurocontrol.int/dashboard/rnd-data-archive, (accessed on 17 January 2024); and from the Reporting Carrier On-Time Performance database of the Bureau of Transportation Statistics, U.S. Department of Transportation, freely accessible at https://www.transtats.bts.gov, (accessed on 17 January 2024). Please note that both data sets have different temporal scopes due to limitations at source: while data are limited for the EU to four months (i.e., March, June, September, and December) of five years (2015–2019), the US dataset includes all months for the same five years. A list of the airports included in the study, alongside some basic statistics on operations, is reported in Table A1 and Table A2. From these raw data, arrival delay time series have been extracted, calculated as the difference between the actual and scheduled landing times of each flight, and averaged at each destination airport and each hour of the day.

It is interesting to note that the two considered data sets are highly heterogeneous. Firstly, as already discussed, the European one does not cover a continuous span of time. Secondly, flights and their associated delays are reported differently [54]: in the US case, flights correspond to those operated by certified US air carriers accounting for at least one percent of domestic scheduled passenger revenues (as opposed to all flights of the European case), and their associated delays are as reported by the airline (as opposed to by a central organization, i.e., EUROCONTROL in the European case).

## 3. Results

We start the analysis of the results by evaluating the fraction of functional links detected in the daily shuffled surrogate time series as a function of the two evaluation metrics and under different procedures for detrending. Please note that, due to the use of surrogate time series, no functional connectivity can be present; hence, those links that are detected represent how much the functional metrics are misled by the residual nonstationarity of the time series—this fraction is therefore called “confusion” in what follows.

**Figure 3:**
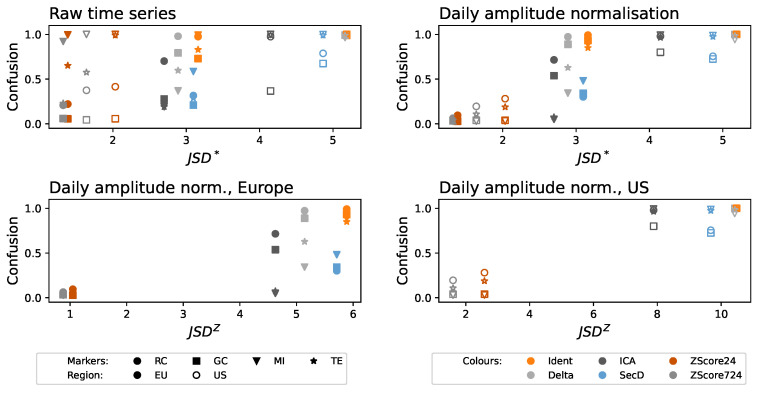
Confusion, i.e., fraction of times a functional relationship is detected between two airports when data are daily shuffled, as a function of the $JSD^{*}$ of the detrended time series. (**Top left and right**) panels respectively correspond to the original time series, and thereto daily normalized in amplitude using a Z-Score—see main text for details. Bottom panels further report the results as a function of the $JSD^{Z}$ for the daily normalized time series, distinguishing between Europe (**bottom left**) and the US (**bottom right**). Shape and colors of the markers respectively indicate the functional metric and the detrending procedure; solid and empty markers further correspond to EU and US, see bottom legends.

**Figure 4:**
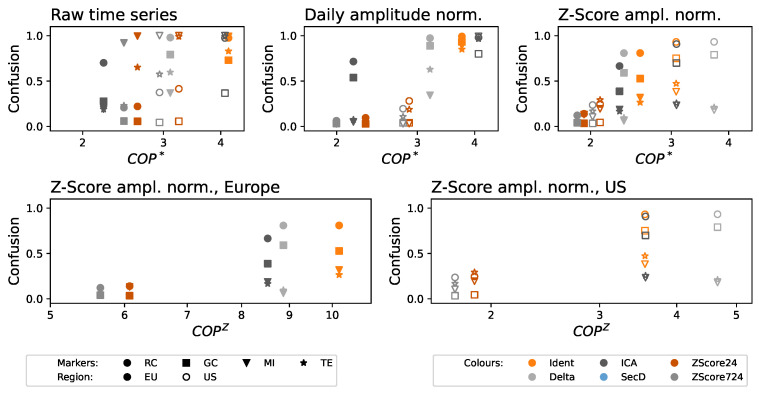
Confusion, i.e., fraction of times a functional relationship is detected between two airports when data are daily shuffled, as a function of the $COP^{*}$ of the detrended time series. **Top** panels respectively correspond to the original time series, and daily normalized in amplitude using a Z-Score, and normalized across airports—see main text for details. **Bottom** panels further report the results as a function of the $COP^{Z}$ for the Z-Score normalized time series, distinguishing between Europe (bottom left) and the US (bottom right). Shape and colors of the markers respectively indicate the functional metric and the detrending procedure; solid and empty markers further correspond to EU and US, see bottom legends. Please note that points corresponding to the SecD (light blue) are not visible due to this procedure yielding values of $COP^{*}>8$ and $COP^{Z}>15$.

The top left panel of Figure 3 reports the evolution of the confusion as a function of the $JSD^{*}$, in a way that will be common to all panels in subsequent figures, e.g., Figure 3 and Figure 4. Specifically, each point represents the result obtained using a combination of detrending strategies and functional connectivity metrics; the former is indicated by the shape of the marker, and the latter by its color—see legends in the bottom part. Please note that, according to the initial hypothesis of this work, the more a time series is detrended, the lower the quantity of information in them detected by the $JSD^{*}$, and, conversely, the lower should be the fraction of times that the functional connectivity metrics become confused by the nonstationarity. In other words, one would expect a positive correlation between the two, or at least no points corresponding to a low $JSD^{*}$ and large confusion. As can be appreciated in Figure 3, this is not the case, with many points being located in the top left quadrant. In other words, even when the permutation pattern-based metric identifies no clear structures in the data (note the minimum $JSD^{*}\approx 1.5$), these still have residual nonstationarity that results in spurious causality relations.

The reason for this negative result is easily identifiable by recalling that permutation patterns are designed to assess the presence of temporal structures in the data at the cost of disregarding the amplitude of the same. To illustrate, the top right panel of Figure 3 reports the same results when the original time series of delays are daily normalized using a Z-Score; in other words, the segment of 24 h corresponding to one day and airport is transformed to have an average of zero and a standard deviation of one. The same results as a function of $JSD^{Z}$, divided according to European and US airports, are reported in the bottom panels of the same figure. An S-shaped curve readily emerged, suggesting that most of the seasonalities are lost using this second normalization process. However, it is worth noting that this assessment is conducted by relying on the detected functional connectivity, i.e., a posteriori.

A solution to this problem may come from the use of a modified version of the permutation pattern paradigm, taking into account the local amplitude structure, i.e., the concept of COP. Figure 4 then reports the results obtained as a function of $COP^{*}$, in a way similar to Figure 3. Specifically, the top left panel reports the confusion as a function of the $COP^{*}$ when networks are reconstructed with the raw time series; the value of $COP^{*}$ remains high, suggesting the presence of residual patterns. The same metric drops in the two following panels. Specifically, the top middle one corresponds to the daily amplitude normalization, i.e., the same as the right panel of Figure 3. Next, the top right panel corresponds to a normalization in which the value of the delay observed at one airport at one given hour is normalized, using a Z-Score, against the set of delays in all other airports at the same time. Please note that this second normalization accounts for the amplitude of delays across the whole system, as opposed to one single airport. As can be seen, in both cases, the value of the $COP^{*}$, at least for the Z-Score detrending methods (brown and gray markers), drops close to or below 2.0; the residual number of functional relationships is also significantly reduced. Results for the latter normalization as a function of $COP^{Z}$, divided according to European and US airports, are reported in the bottom panels of the same figure.

**Figure 5:**
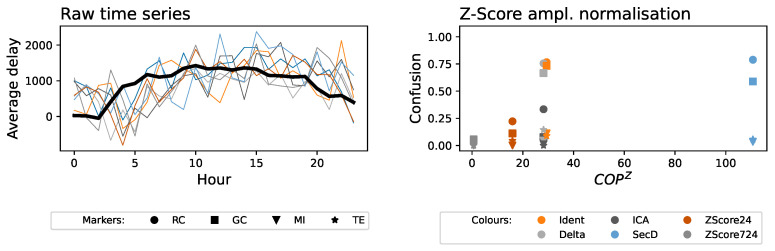
Analysis of synthetic data. The **left** panel reports a representation of the global average delay profile (thick black line) across the 24 h of the day and of one realization of the delay for the seven days of the week (thin colored lines). The **right** panel reports the confusion as a function of the $COP^{Z}$ on the synthetic time series. See the main text for details on the generation process. Shape and colors of the markers respectively indicate the functional metric and the detrending procedure.

In order to confirm the validity of these results, we tested the previous insights using a synthetic dataset constructed by following the expected behavior of delays. We specifically started by calculating the average evolution of the hourly delays at London Heathrow across September 2019; see the solid black line in the left panel of Figure 5. Starting from this, a synthetic time series was generated by adding a noise, drawn from a distribution N(0.0,420.0), that repeated every seven days and an additional noise drawn from a distribution N(0.0,140.0). In other words, the time series of an airport is given by a repeated daily pattern, modulated by a weekly noise of ≈1/3 of the total signal amplitude, and with an additional random component of amplitude ≈1/3 of the previous one. A graphical representation of seven days is included in the left panel of Figure 5; see the thin colored lines. Ten time series, representing the dynamics of ten airports across 30 days, are finally created, each one including a random time shift—to simulate, e.g., the fact that some airports may have their peak time at different hours of the day. Please note that these time series are highly non-stationary (as, indeed, the main component repeats every day) and that, therefore, a functional connection is detected between them if not properly pre-processed. When the same detrending processes are applied to these time series, the results are as expected, see right panel of Figure 5; specifically, the Z-Score_724_ and the Z-Score_24_ are the two best approaches, in agreement with the way data were synthesized. Additionally, the low values of confusion confirm that no false positives are generated.

Given the previous results, one may be tempted to conclude that the solution is clear: the two variants of the Z-Score detrending, in conjunction with the Z-Score amplitude normalization, yield time series with low enough trends. This is, nevertheless, not the full picture, and two additional and complementary aspects must be analyzed: is a $COP^{*}<2$ low enough? As discussed in Section 2.2.4, this value is equivalent to a Z-Score of ≈6, and hence to the presence of structures that are highly statistically significant. Conversely, does this detrending/normalization preserve enough information about the real functional relationships?

**Figure 6:**
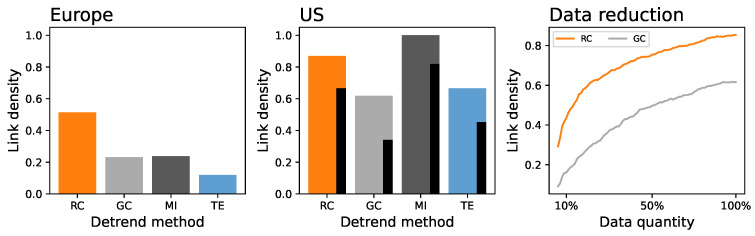
Real functional connectivity. (**Left and center**) Fraction of pairs of time series for which functional connectivity is detected, for Europe (**left panel**) and the US (**central panel**), and four different connectivity metrics. In the latter case, black bars report the link density obtained when US time series are pruned to match European ones. (**Right**) Evolution of the detected link density in the US system as a function of the percentage of data used in the evaluation. Original time series have been processed using a combination of Z-Score_724_ detrending and Z-Score amplitude normalization.

In order to answer these two questions, we calculated the number of functional links obtained when analyzing the real data, i.e., without any daily shuffling, for the four functional metrics, and using the combination of Z-Score_724_ detrending and Z-Score amplitude normalization. Results, reported in the left and central panel of Figure 6, indicate that a large number of functional links are still detected. The case of the US (central panel) is interesting in that a high functional link density is obtained, much higher than in the EU case, with MI even yielding a fully connected network—which may indicate that not all trends are actually deleted. This nevertheless seems to be partly due to the much larger quantity of data available for the US; as described in Section 2.4, these include all months of the five considered years, as opposed to four months per year of the European dataset. When the time series are pruned to match the other dataset’s days, the link density observed for the US substantially drops—see black bars in the central panel of Figure 6. This is, of course, to be expected, as the lower the quantity of available data, the more difficult it is to detect functional relationships, and hence, the lower the obtained link density—see also the right panel of the same figure.

## 4. Discussion and Conclusions

This contribution tackled two complementary questions: whether ordinal patterns can be used to evaluate the stationarity of time series for then using these to assess the suitability of standard detrending methods in the context of air transport delay data. The discussion of the results must, therefore, be addressed from this two-fold perspective.

On the one hand, ordinal patterns seem to be a good quantifier of the quantity of temporal structure in the data and, hence, their nonstationarity. These work better than classical statistical tests [18,19] and may be the solution to some known problems they pose [55]. As frequently discussed in the literature, the original version of this metric disregards the amplitude of the time series and, therefore, cannot detect non-stationarities that are amplitude-based—a problem well-known in the literature [56,57]. This can be improved by resorting to modified versions of the same that include local or global amplitude information. We have here considered Continuous Ordinal Patterns—see Figure 4; the reader should nevertheless take into account that other alternatives exist, including weighted permutation patterns [58], generalized patterns [59], or slope entropy [60]. In synthesis, given a detrending method, this can easily be evaluated using an ordinal-based approach, and this conclusion is of general applicability beyond the specific dataset considered here.

On the other hand, we have evaluated a suite of detrending methods commonly used to reconstruct functional networks representing the propagation of delays in air transport; and what obtained depicts a complex scenario. First, a detrending method alone is not enough to obtain stationary data—see left panels of Figure 3 and Figure 4. This can be explained by taking into account the presence of confounding factors that may affect the observed values, both within the same day and across multiple airports of the system. To illustrate, suppose a day with adverse weather throughout the continent. Delays will be higher than expected, both in a given airport for multiple hours (possibly the whole day) and across all the airports of the affected region. A detrend method alone cannot account for this, and its use would result in many functional links that do not necessarily correspond to true propagations. This problem is easily solved by incorporating an additional normalization of the values, either through time (right panel of Figure 3 and central panel of Figure 4) or through airports (right panel of Figure 4). In other words, the problem at hand requires a multi-variate style detrending, something not common in the literature [61]. It is worth noting that, to the best of our knowledge, the proposed normalizations have hitherto never been applied in this context.

**Figure 7:**
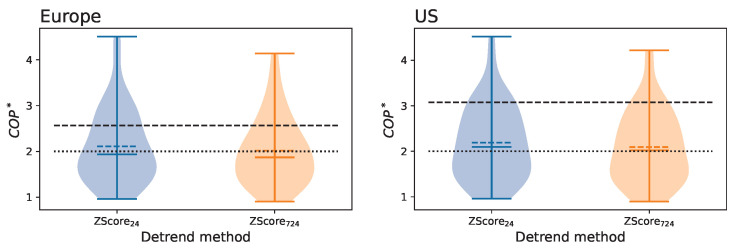
Distribution of the $COP^{*}$ of time series for all airports in Europe (**left panel**) and US (**right panel**), when these are detrended using Z-Score_24_ (left graph, blue) and Z-Score_724_ (right graph, orange) methods, and with a Z-Score amplitude normalization. Within each distribution, the middle horizontal lines indicate the median (solid line) and the mean (dashed line). The dashed black horizontal lines indicate the median value observed in the raw data, i.e., before any detrending/normalization. Finally, the dotted lines correspond to a $COP^{*}=2$ and are included as a visual reference.

On a negative note, we also observed that the use of this combination of detrending/normalization methods is not enough to ensure a complete filtering of the time series. Specifically, the $COP^{*}$, while lower than in the raw time series, is still quite high—see Figure 4 and Figure 7; and this results in a link density >0.1 for daily shuffled time series, and suspiciously high connectivity for the raw data—see Figure 6. This is more prominent in the case of the US. Beyond the availability of more data, we hypothesize that this is a consequence of the larger geographical dispersion of this air transport system and of the presence of multiple time zones, which may hinder the Z-Score normalization across airports. Thus, when considering the problem of air transport delay propagations, the conclusion that can here be drawn is that better (possibly tailored) methods for data detrending are needed.

It is worth noting that some detrending approaches, which are common in the literature, yield results that are actually worse than performing no pre-processing—see, for instance, the cases of SecD in Figure 3 and ICA in Figure 4, respectively yielding higher $JSD^{*}$ and $COP^{*}$. This may be due to underlying hypotheses that are not fulfilled by the system under analysis. To illustrate, ICA expects components in the signals to be synchronized across all of them [26]; yet, rush hours at airports may be different, especially when these are located in different time zones.

As a final point, some interesting operational conclusions can be drawn from Figure 6. Even when pruning the time series to include the same number of days, higher link densities are obtained for the US; in addition, this difference is especially notable for MI and TE. This indicates that the US air transportation system is more prone to propagate delays, possibly in a more non-linear and complex way. Please note that this may be caused by multiple factors. To illustrate, the EU strongly relies on strategic Air Traffic Flow Management (ATFM) regulations, which aim at avoiding airborne holdings, while the US equivalent Ground Delay Program (GDP) works on shorter time scales [62]. Additionally, delays are reported in slightly different ways, as these are calculated in Europe using the last-filed flight plans and thus already contain strategic ATFM adjustments [54]. Lastly, even the origin of the data may have an impact: while in Europe, delays are calculated by a central authority, in the US, they are reported by individual airlines, which may follow different standards for their estimation.

In conclusion, the detrending of real-world time series has been shown to be a complex problem in which the idiosyncrasies of the studied system have a non-negligible impact on the validity of the process. Ordinal patterns-based metrics can nevertheless be used to validate the detrending process by quantifying the remaining amount of temporal structures.

## Data Availability

No new data were created or analyzed in this study. The original data analyzed in the study are openly available at https://www.eurocontrol.int/dashboard/rnd-data-archive (accessed on 13 July 2023) and https://www.transtats.bts.gov (accessed on 24 February 2024).

## Appendix A

**Table A1:** Information about European airports. As well as ICAO codes and official names, the table includes the average number of hourly departures and arrivals using the operations available in the dataset.

Rank	Code	Name	Departures	Arrivals
1	EGLL	Heathrow Airport	26.39	26.41
2	LFPG	Charles de Gaulle Airport	26.97	26.91
3	EHAM	Amsterdam Airport Schiphol	27.37	27.31
4	EDDF	Frankfurt am Main Airport	26.92	26.89
5	LEMD	Adolfo Suárez Madrid-Barajas Airport	21.93	21.90
6	LEBL	Josep Tarradellas Barcelona-El Prat Airport	17.77	17.76
7	EDDM	Munich Airport	22.13	22.14
8	EGKK	Gatwick Airport	15.83	15.79
9	LIRF	Leonardo da Vinci-Fiumicino Airport	17.17	17.17
10	LFPO	Orly Airport	12.96	12.94
11	EIDW	Dublin Airport	12.24	12.22
12	LSZH	Zurich Airport	14.15	14.11
13	EKCH	Copenhagen Airport	14.62	14.64
14	LEPA	Palma de Mallorca Airport	10.97	10.99
15	LPPT	Lisbon Airport	11.00	11.01
16	ENGM	Oslo Airport, Gardermoen	13.75	13.75
17	EGCC	Manchester Airport	10.84	10.82
18	EGSS	London Stansted Airport	10.01	10.00
19	LOWW	Vienna International Airport	13.91	13.89
20	ESSA	Stockholm Arlanda Airport	13.31	13.31
21	EBBR	Brussels Airport	12.54	12.51
22	LIMC	Malpensa Airport	10.49	10.49
23	EDDL	Düsseldorf Airport	11.96	11.94
24	LGAV	Athens International Airport	10.62	10.63
25	EDDT	Berlin Tegel Airport	10.18	10.17
26	LEMG	Málaga Airport	6.82	6.82
27	EPWA	Warsaw Chopin Airport	9.21	9.21
28	LSGG	Geneva Airport	9.63	9.63
29	EDDH	Hamburg Airport	8.16	8.16
30	LKPR	Václav Havel Airport Prague	7.80	7.81
31	EGGW	Luton Airport	6.84	6.85
32	LHBP	Budapest Ferenc Liszt International Airport	5.78	5.77
33	EGPH	Edinburgh Airport	6.87	6.86
34	LEAL	Alicante-Elche Airport	4.94	4.94
35	LFMN	Nice Côte d’Azur Airport	7.22	7.23
36	LROP	Henri Coanda International Airport	6.20	6.19
37	EDDK	Cologne Bonn Airport	7.27	7.26
38	LIME	Orio al Serio International Airport	4.69	4.68
39	UKBB	Boryspil International Airport	4.88	4.89
40	EGBB	Birmingham Airport	6.01	6.00
41	LPPR	Porto Airport	4.73	4.73
42	EDDS	Stuttgart Airport	6.45	6.45
43	LIPZ	Venice Marco Polo Airport	4.99	4.99
44	LFLL	Lyon-Saint-Exupéry Airport	6.30	6.30
45	LICC	Catania-Fontanarossa Airport	3.66	3.66
46	LIRN	Naples Airport	3.85	3.85
47	EGPF	Glasgow Airport	4.86	4.84
48	LFBO	Toulouse-Blagnac Airport	5.14	5.11
49	LFML	Marseille Provence Airport	5.21	5.20
50	LIML	Linate Airport	5.88	5.88

**Table A2:** Information about European airports. As well as IATA codes and official names, the table includes the average number of hourly departures and arrivals using the operations available in the dataset.

Rank	Code	Name	Title 3	Rank
1	ATL	Hartsfield-Jackson Atlanta International Airport	41.63	41.61
2	DEN	Denver International Airport	25.41	25.38
3	DFW	Dallas Fort Worth International Airport	25.42	25.35
4	LAX	Los Angeles International Airport	23.76	23.77
5	ORD	Chicago O’Hare International Airport	31.42	31.37
6	PHX	Phoenix Sky Harbor International Airport	17.81	17.79
7	MSP	Minneapolis-Saint Paul International Airport	14.95	14.95
8	CLT	Charlotte Douglas International Airport	16.17	16.14
9	SEA	Seattle-Tacoma International Airport	14.88	14.88
10	SFO	San Francisco International Airport	18.77	18.77
11	JFK	John F. Kennedy International Airport	11.69	11.68
12	IAH	George Bush Intercontinental Airport	17.04	17.00
13	MCO	Orlando International Airport	14.51	14.51
14	EWR	Newark Liberty International Airport	13.48	13.46
15	LAS	Harry Reid International Airport	17.10	17.12
16	FLL	Fort Lauderdale-Hollywood International Airport	9.87	9.87
17	BOS	General Edward Lawrence Logan International Airport	14.34	14.36
18	DTW	Detroit Metropolitan Wayne County Airport	14.45	14.47
19	MIA	Miami International Airport	8.31	8.31
20	LGA	LaGuardia Airport	12.84	12.80
21	IAD	Washington Dulles International Airport	5.37	5.37
22	BWI	Baltimore/Washington International Thurgood Marshall Airport	10.96	10.96
23	PHL	Philadelphia International Airport	9.53	9.53
24	SAN	San Diego International Airport	9.28	9.28
25	MDW	Chicago Midway International Airport	9.45	9.43
26	SLC	Salt Lake City International Airport	11.91	11.93
27	DCA	Ronald Reagan Washington National Airport	10.32	10.32
28	TPA	Tampa International Airport	7.89	7.90
29	PDX	Portland International Airport	6.58	6.59
30	STL	St. Louis Lambert International Airport	6.47	6.48
31	BNA	Nashville International Airport	6.78	6.79
32	AUS	Austin-Bergstrom International Airport	6.04	6.05
33	HNL	Daniel K. Inouye International Airport	5.44	5.44
34	SJC	San José International Airport	5.50	5.50
35	MCI	Kansas City International Airport	5.24	5.25
36	DAL	Dallas Love Field	7.65	7.63
37	SMF	Sacramento International Airport	4.97	4.98
38	MSY	Louis Armstrong New Orleans International Airport	5.40	5.40
39	SNA	John Wayne Airport	4.56	4.57
40	RDU	Raleigh-Durham International Airport	4.88	4.89
41	RSW	Southwest Florida International Airport	3.42	3.42
42	PIT	Pittsburgh International Airport	3.78	3.79
43	HOU	William P. Hobby Airport	6.17	6.16
44	IND	Indianapolis International Airport	3.75	3.76
45	SAT	San Antonio International Airport	3.87	3.88
46	SJU	Luis Mu noz Marín International Airport	2.84	2.85
47	CLE	Cleveland Hopkins International Airport	4.36	4.37
48	OAK	Oakland International Airport	5.54	5.53
49	CVG	Cincinnati/Northern Kentucky International Airport	3.10	3.11
50	CMH	John Glenn Columbus International Airport	3.44	3.45
